# Object state optimization algorithm based on Bayesian random sampling for visual object tracking

**DOI:** 10.1038/s41598-025-21033-2

**Published:** 2025-10-24

**Authors:** Zhiqiang Zhao, Huijie Zhao, Daitu Wen, Tao Ma, Xiaoli Luo, Bin Wu

**Affiliations:** 1 The School of Mathematics and Computer Science, Ningxia Normal University, Guyuan, Ningxia 756099 People’s Republic of China; 2 Artificial Intelligence and Intelligent Medical Engineering Technology Research Center, Guyuan, Ningxia 756099 People’s Republic of China; 3 The School of Information Science and Technology, University of Jiujiang, Jiujiang, Jiangxi 332005 People’s Republic of China

**Keywords:** Visual object tracking, State estimation, Dense sampling, Bayesian random sampling, Motion detection, Object vision

## Abstract

From the perspective of object state modeling, visual object tracking can be regarded as a unified process that combines object state estimation and object localization. In this framework, state estimation refers to predicting the complete state vector of the object–such as its position, scale, and motion dynamics–while localization specifically denotes identifying the object’s spatial position within the image, typically in the form of bounding box coordinates. Traditional optimization-based methods for state estimation often suffer from getting trapped in local optima, primarily due to the non-convexity of the objective function and the algorithm’s sensitivity to initialization. To address these issues, this research proposes an object state optimization algorithm based on Bayesian random sampling for visual object tracking. Firstly, a dense sampling method is introduced to mitigate the problem of local optima. Secondly, a hybrid model that merges Bayesian random sampling and gradient ascent is proposed to refine the bounding box, successfully alleviating convergence instability. Finally, our experimental results show that the proposed algorithm significantly improves tracking performance on multiple datasets, validating its efficiency and applicability in object state estimation tasks.

## Introduction

Object tracking plays a crucial role in a wide range of application scenarios, with the aim of accurately and stably estimating the position and state of objects within a sequence^1,2^. In recent years, significant advancements have been made in object tracking technology within the field of computer vision, such as remote sensing satellites^3^ and unmanned aerial vehicles (UAV)^4^. However, due to factors such as illumination variations and occlusions, object tracking remains an inherently challenging task.

Currently, deep learning-based object tracking methods^1,5^ have primarily been categorized into four types: Siamese network-based architectures^6,7^, discriminative model-based architectures^8,9^, Transformer-based architectures^10,11^, and multi-technology fusion architectures^12,13^. Siamese network-based trackers correlate object template features with search region convolutional features, trained end-to-end on datasets^14,15^. Discriminative model-based methods employ a discriminator to distinguish between object and background information, achieving accurate tracking by predicting the object’s bounding box^16^. Transformer-based approaches emphasize the use of self-attention and cross-attention mechanisms for object tracking and localization^17,18^. Multi-technology fusion-based algorithms emphasize the integration of diverse techniques or models to complement each other, achieving high-precision and robust object tracking^19,20^. These object tracking algorithms focus on object classification or state estimation as the core for analyzing and tracking objects. Object state estimation, as a key step in object tracking, aims to further refine the object’s location and optimize the bounding box based on the initial detection results of the object. In order to achieve more accurate object state estimation, researchers have proposed various strategies to optimize this process. These algorithms can be broadly divided into two categories based on their design philosophies: one category integrates object localization and bounding box parameter regression into a unified model for joint optimization^21^, while the other models them separately in a staged manner^22^. On the one hand, some researchers estimate the object bounding box by first performing object center point localization or keypoint localization and then deducing the bounding box parameters. For example, Hui et al.^23^ utilize Vision Transformer (ViT) and the Template-Bridged Search Region Interaction (TBSI) module to extract spatio-temporal features, predicting the object position and subsequently inferring the bounding box. Talaoubrid et al.^22^ employ particle filtering to approximate the object state probability distribution and estimate the bounding box accordingly. Zhao et al.^7^ convert 3D point cloud data into 2D feature maps via BEV and use convolutional neural networks (CNN) combined with supervised by a loss function to predict object position for bounding box estimation. These methods improve robustness and accuracy to some extent. However, due to real-time constraints, they show limited precision in accurately predicting object bounding boxes. On the other hand, some researchers focus on directly optimizing bounding box parameters to address real-time limitations in object tracking. Models such as Siamese-RPN^24^, ATOM^25^, DiMP^26^ and SeqTrack^27^ have effectively enhanced tracking robustness and accuracy. Specifically, ATOM^25^ combines a classifier with an IoU predictor and uses gradient ascent to optimize candidate box parameters, alleviating the classification-localization inconsistency in traditional tracking methods. DiMP^26^ further introduces a discriminative filter and employs steepest descent in continuous space to iteratively search for the optimal bounding box, enabling end-to-end training and fast online updating. These tracking methods have achieved good results in object state estimation, improving tracking performance and accuracy while alleviating real-time constraints to a certain extent. However, there are still some challenges. First, due to non-convex loss functions and dependence on initialization, they are prone to local optima and fail to reach the global optimum within limited time. Second, during the optimization process, pronounced oscillations often occur near the optimal solution region. This is mainly caused by unstable gradients and ambiguous feature responses, leading the solution to fluctuate repeatedly among multiple approximate optima, making convergence unstable.

To address the above challenges, this paper proposes an object state optimization algorithm based on Bayesian random sampling for visual object tracking(BRSO), aiming to capture the optimal state information of the tracked object. Firstly, a dense sampling method is introduced to help the model overcome the limitations of conventional optimization algorithms, enabling it to escape the constraints of local optima. Secondly, to address oscillations in the predicted object state values, a hybrid model combining gradient ascent and Bayesian random sampling principles is developed. The core of this approach lies in leveraging the random sampling principle to enhance the diversity and randomness of candidate bounding boxes, while maximum a posteriori (MAP) estimation improves the accuracy of the predicted object state information. Additionally, IoU features are introduced as key metrics for optimising and predicting object bounding boxes, with extensive experiments across various datasets and tracking methods. The results show that the method significantly improves the object tracking performance and verify its efficiency and applicability in the task of object state estimation.

The main contribution of this paper is:

1. Proposed a dense sampling method aimed at overcoming the iterative limitations of traditional optimization algorithms, enabling global optimization of the model.
2. Introduced a hybrid optimization strategy based on Bayesian random sampling principles and the gradient ascent algorithm to mitigate oscillation issues in object state prediction, resulting in more precise object bounding boxes.
3. Validated the effectiveness and compatibility of the proposed method on multiple datasets. Experimental results demonstrate that the method achieves outstanding performance in object tracking state estimation tasks.

## Relevant work

### IoU

In recent years, the IoU feature has been widely used as a key metric for measuring bounding box overlap in visual perception and prediction systems. IoU is defined as the ratio between the overlapping area and the union area of two bounding boxes, with values ranging from 0 to 1. Its applications include bounding box regression loss optimization in object detection^28,29^; trajectory matching and candidate box quality evaluation in visual tracking^30^; multi-model detection result fusion and duplicate removal^31^; as well as object state estimation^32^.

To improve the accuracy of object state estimation, a variety of studies have explored integrating and extending IoU features. Overall, these methods can be categorized into the following three types:

**Joint Modeling of IoU Features with Spatio-Temporal Information:** These methods combine IoU features with appearance, motion, and other Spatio-Temporal features as joint inputs to the model^33,34^. For example, NeighborTrack^34^ incorporates IoU as a spatial location feature into a bipartite graph-based matching framework, enhancing object matching discrimination and stability. However, such approaches suffer from convergence instability due to feature conflicts and neighbor dependency.

**Introducing Spatial Uncertainty Modeling Mechanisms:** These methods introduce spatial uncertainty evaluation into IoU-based state estimation to refine bounding box estimation^35–37^. For instance, SSUTracker^37^ improves the robustness and accuracy of bounding box estimation by combining weighted GIoU with spatial uncertainty scores to predict more accurate object positions. Nevertheless, IoU as a distance metric provides limited information, making it difficult to handle complex scenarios.

**State Estimation Mechanisms Based on IoU Prediction and Fusion:** These methods explicitly or implicitly use *IoU* prediction results to optimize object bounding boxes^25,26,38–41^. For example, PrDiMP^40^ extends DiMP by incorporating KL divergence to model the distribution of IoU predictions, mitigating fluctuations caused by single-point predictions. However, its static single-step optimization structure struggles to dynamically adapt to spatio-temporal variations in object states. KYS^41^ implicitly integrates IoU-equivalent spatial overlap features through state vector propagation to achieve object region discrimination and localization optimization. Yet, its state updating depends heavily on previous frame estimations, leading to error accumulation and amplification.

In summary, existing methods have improved the accuracy of bounding box prediction to a certain extent. However, due to the non-convexity of the loss function and dependency on initial bounding boxes, there remains a risk of falling into local optima. To address this issue, our method takes IoU features as the primary metric for object state optimization and prediction, introducing a dense sampling strategy combined with sample diversity modeling mechanisms to generate high-quality candidate boxes.

### Object bounding box estimation

Inspired by object detection methods^42^, SiamRPN^24^ and its extension SiamRPN++^43^ adopt an RPN-based regression framework, using classification and regression branches with anchor boxes to predict object position and size. While these methods demonstrate strong performance, the decoupling between classification and regression leads to suboptimal matching when handling large scale variations or objects with irregular shapes. To overcome the limitations of anchor-based designs, anchor-free approaches such as SiamBAN^44^, SiamCAR^45^, and DEST^46^ utilize fully convolutional networks (FCN) to estimate object confidence scores and predict bounding boxes. These methods improve prediction accuracy to some extent, but still suffer from regression instability. ATOM^25^ and DiMP^26^ further extend anchor-free strategies by introducing *IoU* prediction networks for posterior gradient-based optimization. By averaging multiple high-IoU candidates, these methods refine bounding box estimation. However, gradient instability remains a challenge during the parameter search process, affecting the consistency and convergence of results. In recent years, Transformer-based models have been widely applied for direct bounding box regression^27,47–54^. For example, Trtr^47^ employs a Transformer encoder–decoder structure, TransT^49^ integrates ResNet-50 with attention mechanisms, and SeqTrack^27^ predicts bounding box sequences through an end-to-end autoregressive method. These approaches effectively leverage temporal information to enhance prediction performance. Nevertheless, due to model complexity and real-time constraints, achieving stable convergence remains challenging. To further improve bounding box quality, E.T.Track^55^ and IoUformer^32^ incorporate *IoU* prediction networks into Transformer-based tracking frameworks. By filtering confidence scores and refining bounding box regression, they enhance prediction accuracy. However, constrained by optimization complexity and real-time requirements, the models still suffer from convergence instability. In summary, the aforementioned tracking algorithms have improved tracking performance and robustness to a certain extent. However, due to the limitations of existing optimization algorithms, they are limited in efficiently reaching global optima within limited iterations, often exhibiting oscillation phenomena near optimal solutions. As a result, the predicted bounding boxes may not fully reflect the actual object boundaries. To address these issues, this paper proposes a hybrid model that integrates gradient ascent^56^ with Bayesian random sampling^57^ to optimize object state estimation through dense sampling.

**Fig. 1:**
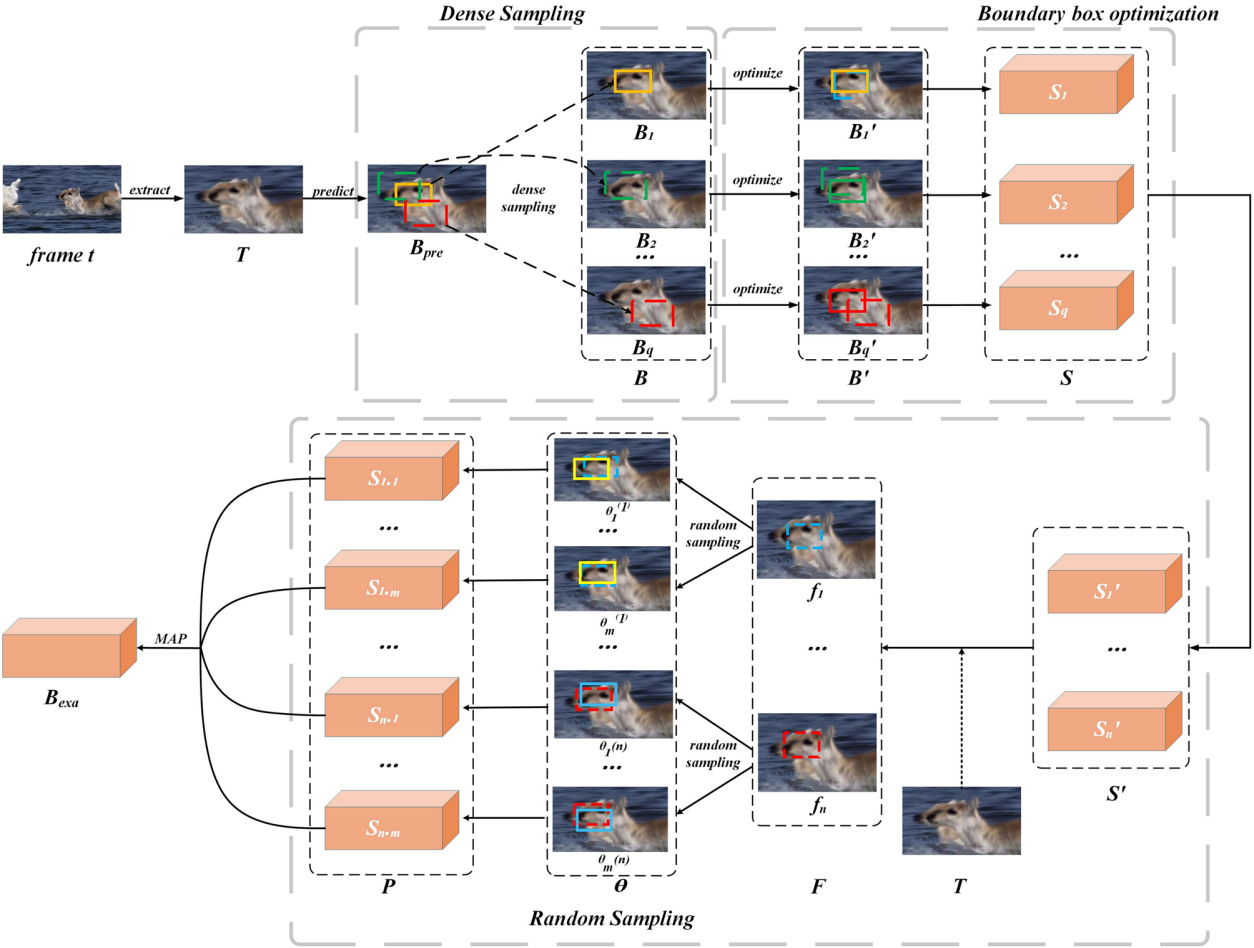
Object state optimization algorithm based on Bayesian random sampling for visual object tracking. Starting with the preliminary predicted object state $B_{pre}$, an object state set $B^{'}$ is generated through dense sampling, and each object state is optimized using gradient ascent. Then, *n* best states are selected, Bayesian random sampling is performed, and the final optimal object state $B_{exa}$ is determined using the maximum a posteriori probability.

## Proposed method

The process of visual object tracking is divided into two stages: object localization and bounding box optimization. To clearly describe the proposed bounding box optimization algorithm, we assume that the prediction network obtains the object prediction spectrogram *M* during the object localization phase, and preliminary predictions for the object state are $B_{t}$, $B_t=(C,W,H)$, where *C* denotes the center coordinates, *W* denotes the width, and *H* denotes the height. $M_{B_{t}}$can be expressed as:

1 $$\begin{aligned} M_{B_{t}} = g\big (a(X_{0},B_{0}),z(X_{t},B_{t-1})\big ), \end{aligned}$$

Here, *a* represents the reference vector of the object, derived from $X_{0}$ and $B_{0}$, where $X_{0}$ denotes the backbone features extracted from frame 0, and $B_{0}$ represents the object state in frame 0. *z* denotes the modulation vector computed from $X_{t}$ and $B_{t-1}$, while *g* is the prediction network that estimates the object state based on the reference vector and the modulation vector.

To achieve more accurate prediction of the object state, the paper proposes an optimization algorithm based on Bayesian random sampling, to further refine $B_{t}$ building upon the predicted spectrogram *M*. The specific optimization process is as follows: First, starting from the preliminarily predicted object state $B_{pre}$, dense sampling is performed to generate a set of candidate object states, and obtain the set of confidence scores for the states through the IoU network. Then, a few-step gradient ascent algorithm is used to precisely optimize the confidence scores of the object states, enhancing the accuracy of every state evaluations and selecting several optimal object states. Finally, Bayesian random sampling is applied to the optimized object state set to generate a randomly sampled state set. Based on this, the maximum posterior probability rule is used to obtain the exact object state. The detailed process is illustrated in Fig. 1.

### Dense sampling

Based on the given object bounding box, traditional object state optimization algorithms tend to cause the bounding box state $B_{t}$ at frame *t* to fall into a local optimum during the initial sampling optimization phase. To address this issue, we propose a dense sampling method that increases sample diversity and effectively achieves global optimization. To better describe the process of dense sampling, we define the object state space as $\mathbb {R}^4$, the preliminary bounding box $B_{pre}= (x/w, y/h, \log w, \log h)$ in frame *t*, where (*x*, *y*) represents the center coordinates of the bounding box, and (*w*, *h*) represent its width and height, respectively. In this process, based on the preliminary predicted bounding box $B_{pre}$, the random perturbations are applied to sample the centroid coordinates and the dimensions of the bounding box separately. In the sampling process, the dimensions of the bounding box height and width remain unchanged.

We utilize a center-point perturbation method that samples *q* center points around the center on predefined mesh points, and define the bounding box scale factor *s* that determines the interval density of the predefined mesh. The sampling formula for center coordinates $C(x_i, y_i)$ can be expressed as:

2 $$\begin{aligned} \begin{aligned} x_{i}&=x/w+o_{x_{i}} \cdot s_{x} , \, \\ y_{i}&=y/h+o_{y_{i}} \cdot s_{y} , \end{aligned} \end{aligned}$$

where, $o_{x_{i}},o_{y_{i}}\in \{-1,0,1\}$. We perform random sampling of the width and height of the bounding box based on the approximate square side length $l=\sqrt{w \cdot h}$ of the initial bounding box, which is done to improve the robustness of the algorithm to variations in bounding box dimensions and enhance sample diversity. The width and height of the bounding box after random sampling are expressed as:

3 $$\begin{aligned} \begin{aligned} w_{i}&=\log w+r_{w_{i}} , \, \\ h_{i}&=\log h+r_{h_{i}} , \end{aligned} \end{aligned}$$

where, $r_{w_{i}},r_{h_{i}}$ are random variables sampled from a uniform distribution $[-0.5\delta l,0.5\delta l ]$, and $\delta$ is a predefined perturbation ratio. Through the aforementioned sampling of center points and bounding box size, can be generated a dense sampling set $B=\{B_{1},B_{2},\ldots ,B_{q}\}$.

### Optimization of bounding box

For simplicity, this paper utilizes the IoU-prediction network^25^ to generate confidence scores based on $B_{i}$ for optimizing object candidate bounding boxes. The objective is to maximize the confidence scores predicted by the IoU-network, and the objective function is as follows:

4 $$\begin{aligned} S_{i}=IoU(B_{i}). \end{aligned}$$

To enhance the accuracy and reliability of the object candidate boxes, we employ a stochastic gradient ascent-based method^58^ to optimize the confidence scores of the object states. Through a limited number of iterative optimizations, the positions and sizes of each object bounding box in the sample set *B* are progressively refined to maximize the degree of alignment between the object states and the actual object.

We utilize gradient updates to iteratively adjust the parameters of the bounding box. In each iteration, the gradient of the IoU-prediction network is calculated for each candidate bounding box to update the object parameters. The formula is as follows:

5 $$\begin{aligned} B_{i}^{(j+1)}=B_{i}^{(j)}+\alpha _{j} \cdot \bigtriangledown IoU(B_{i}), \end{aligned}$$

Here, $B_{i}^{(j)}$ represents the result of the $i-th$ sample state at the $j-th$ iteration, $\alpha _{j}$ is the learning rate, $\nabla IoU( B_{i})$ represents the gradient of the objective function. The gradient components of IoU with respect to $B_{i}$ are expressed as:

6 $$\begin{aligned} \bigtriangledown IoU(B_{i})=(\frac{\partial IoU}{\partial (x_{i}/w_{i})},\frac{\partial IoU}{\partial (y_{i}/h_{i})},\frac{\partial IoU}{\partial \log w_{i}},\frac{\partial IoU}{\partial \log h_{i}}). \end{aligned}$$

The entire optimization process undergoes $\lambda$ iterations, with the bounding box from each iteration serving as the starting point for the subsequent optimization. The set of object state samples after a limited number of optimization steps is denoted as $B^{'}=\{ B_{1}^{'},B_{2}^{'},\ldots ,B_{q}^{'}\}$.

### Random sampling

The gradient ascent optimization method causes the prediction of the object to gradually approach the optimal solution. However, two issues arise: first, it often requires a lengthy optimization step; second, in the later stages of optimization, it tends to oscillate near the optimal solution. To address these challenges, the BRSO method employs fewer optimization steps to quickly approximate the optimal solution for object prediction and obtain an initial prediction of the object. Subsequently, Bayesian random sampling is employed to refine the object state estimation for improved accuracy.

In the case of obtaining the set of object state samples $B^{'}=\{B_{1}^{'},B_{2}^{'},\ldots ,B_{q}^{'}\}$, we select *n* optimal states from the sample set as priors for prediction, $F=\{f_{1},f_{2},\ldots ,f_{n}\},1<n<q$, where *n* represents the number of object states. For each element $f_{i}$ in the state set, we first generate *m* new object states, denoted as $\Theta =\{\theta _{1},\theta _{2},\ldots ,\theta _{m}\}$. To enhance the diversity of the object states, Gaussian noise is added to the object states, specifically formulated as:

7 $$\begin{aligned} \theta _{j}=f_{i}+N_{j}(c_{x},c_{y},d_{w},d_{h}),1<j< m , \end{aligned}$$

Here, $N_{j}$ represents the four-dimensional Gaussian noise added to the $j-th$ sample, $(c_{x},c_{y})$, $(d_{w},d_{h})$ respectively represent position noise and size noise. The noise in each dimension follows a Gaussian distribution $G(\mu ,\sigma ^{2})$, $\mu$, $\sigma ^{2}$ denotes the mean and variance of the Gaussian noise. Finally, the posterior probability of the new object state is obtained using Bayes’ theorem^57^ with the prior samples, expressed as:

8 $$\begin{aligned} p(f_{i}|\theta _{j}^{(i)})=\frac{p(\theta _{j}^{(i)}|f_{i})p(f_{i})}{p(\Theta )}, \end{aligned}$$

Since all elements in the set $\Theta$ have the same prior probability, the posterior probability is given by:

9 $$\begin{aligned} p(f_{i}|\theta _{j}^{(i)})=\frac{p(\theta _{j}^{(i)}|f_{i})}{\sum _{i=1}^{n}{p(\theta _{j}^{(i)}|f_{i})}}, \end{aligned}$$

Therefore, for all object states in the set *F*, the posterior probability set $E=\{p(f_{1}|\theta _{1}^{(1)}),\ldots ,p(f_{1}|\theta _{m}^{(1)}),\ldots , p(f_{n}|\theta _{1}^{(n)}),\ldots ,p(f_{n}|\theta _{m}^{(n)})\}$ can be obtained by applying the Bayes’ theorem to randomly sampled object states.

The values of the posterior probability score set *P* are obtained using the Maximum A Posteriori (MAP) criterion:

10 $$\begin{aligned} \begin{aligned} \hat{\theta }&=\underset{\theta }{arg\max }\{p(\theta _{1}^{(1)}|f_{1}),\ldots ,p(\theta _{m}^{(1)}|f_{1}),\ldots , p(\theta _{1}^{(n)}|f_{n}),\ldots ,p(\theta _{m}^{(n)}|f_{n})\}. \end{aligned} \end{aligned}$$

## Experimentation

The experiments were conducted using Python and the PyTorch framework on an NVIDIA 2080 GPU. The ResNet-18 architecture was selected as the backbone network, and the proposed optimization method was integrated and validated. The optimization method, BRSO, was integrated into representative object tracking algorithms, including KYS^41^, Dimp^26^, PrDiMP^40^, and SuperDiMP for comparative analysis. Furthermore, the experimental results are further analyzed in depth on the four publicly available datasets: OTB-100^59^, UAV123^60^, VOT2018^61^, and Temple-color-128^62^.

Based on the randomness of the tracking, the average of the results from three runs is reported. In this experiment, 9 dense bounding boxes were selected, and 10 iterations of optimization were performed for each dense sample, with an initial learning rate $\alpha$ set to 0.01. Three optimal states were selected based on the optimization results, and 50 random sample boxes were generated for each optimal state. Each random sample added Gaussian noise, which follows a two-dimensional Gaussian distribution $G\left( 0.8,0.18^2 \right)$

### Quantitative analysis

**Fig. 2:**
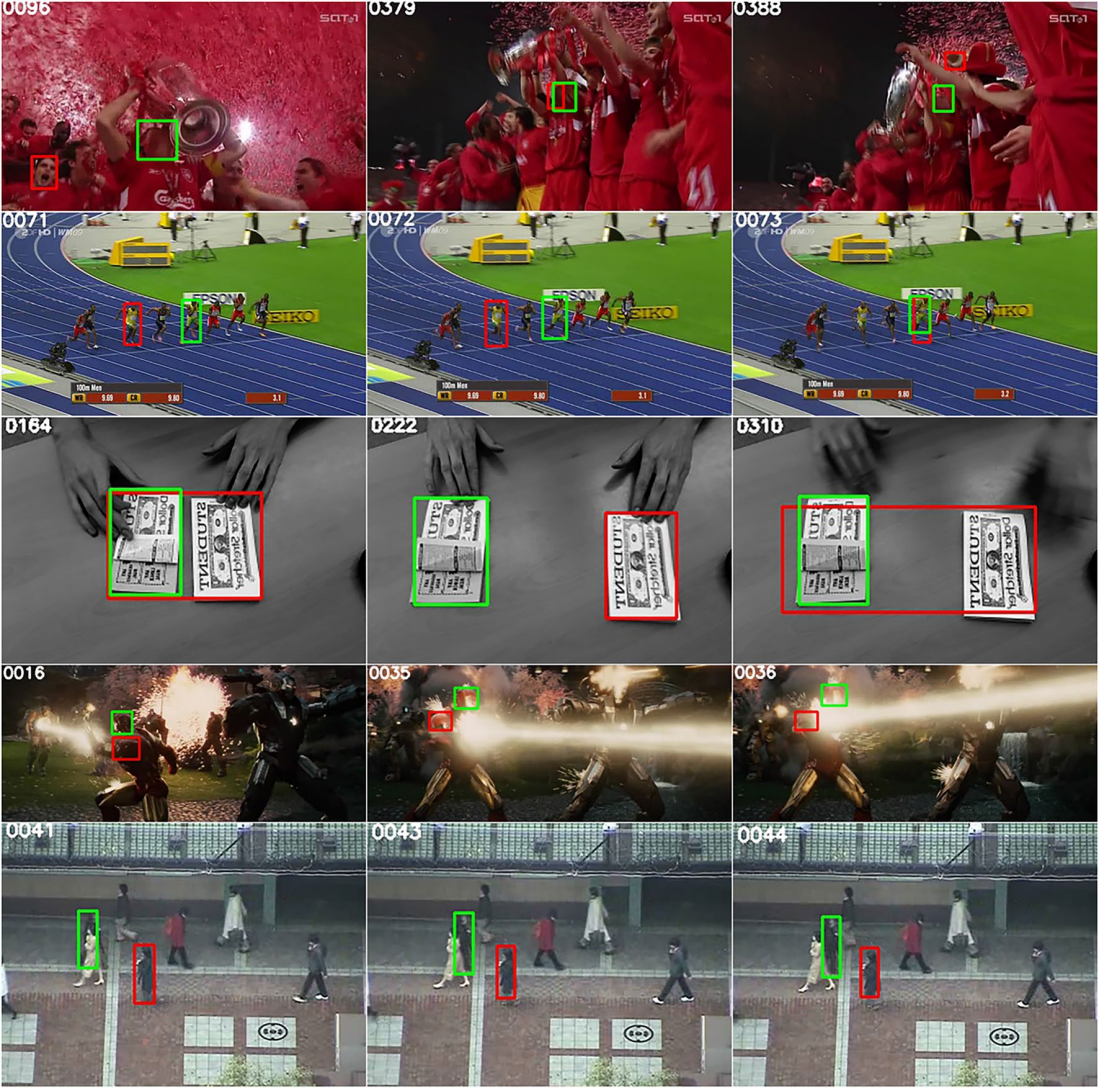
Tracking results on representative sequences from the OTB-100 dataset^59^, including Soccer, Bolt, Coupon, Ironman, and Subway. All images are from this public benchmark. The SuperDiMP tracker is used as the baseline tracker, and BRSO optimization is introduced to enhance the object tracking results. Red indicates the baseline results, while green represents the tracking results after integrating SuperDiMP+BRSO.

To validate the effectiveness of the proposed BRSO algorithm, five representative sequences from the OTB-100 benchmark^59^ –Soccer, Bolt, Coupon, Ironman, and Subway–were selected to evaluate and compare the tracking performance of the baseline SuperDiMP algorithm and the BRSO-enhanced SuperDiMP (SuperDiMP+BRSO). In the SuperDiMP algorithm, IOU features and the steepest descent method are used to select the top three candidates with the highest IoU, and their average is taken to estimate the object state. In this study, SuperDiMP+BRSO replaces this with a MAP-based object state estimation using Bayesian random sampling. Figure 2 illustrates examples of tracking results on the OTB-100 dataset. In the Bolt sequence, fast motion and continuous scale changes, combined with interference from similar objects, cause SuperDiMP to update delayed and inaccurate state updates. Similarly, in the Coupon sequence, distractor objects lead to state estimation failures. After integrating BRSO, both Bolt and Coupon sequences exhibit notably improved object state estimation and tracking accuracy, indicating that the proposed method possesses strong adaptability in handling complex scenarios. Furthermore, in the Soccer sequence, a combination of lighting changes, motion blur, and occlusion causes SuperDiMP to generate unstable object state predictions. In the Ironman sequence, drastic fluctuations in lighting intensity result in SuperDiMP misestimating the object state, mistakenly identifying the shoulder region as the object. In the Subway sequence, when the walking object is occluded, SuperDiMP tends to misidentify similar objects as the object, leading to tracking failure. By comparison, SuperDiMP+BRSO demonstrates consistently superior tracking performance across the Soccer, Ironman, and Subway sequences. This improvement is primarily attributed to the Bayesian random sampling mechanism introduced by BRSO, which enhances both the diversity and discriminative ability of candidate states. In summary, the BRSO optimization strategy enhances both the accuracy and robustness of the tracker under complex scenarios, confirming its effectiveness and applicability in visual object tracking.

### Qualitative analysis

This section presents a qualitative analysis of the proposed algorithm on the OTB-100^59^, UAV123^60^, VOT2018^61^, Temple-color-128^62^ datasets. Success rate is adopted as the primary evaluation metric, while precision and normalized precision are also reported for comprehensive performance.

**Fig. 3:**
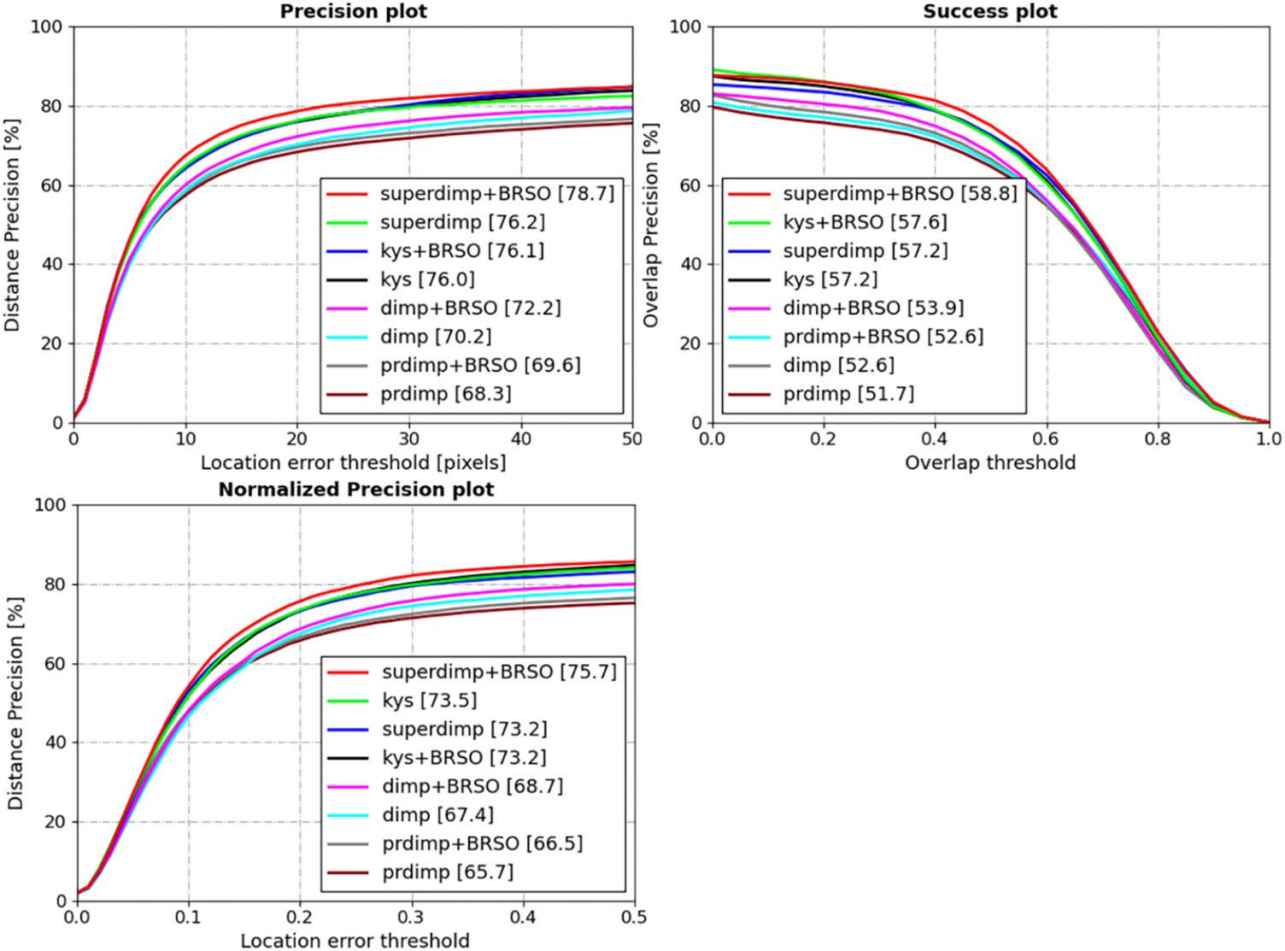
Precision, success rate, and normalized precision evaluation results of various algorithms on the VOT 2018 dataset.

**VOT2018**^61^: The VOT2018 dataset, released in 2018, consists of 60 video sequences encompassing diverse scenarios and challenges, including indoor, outdoor, daytime, and nighttime conditions. On the VOT2018 dataset, we integrated the proposed BRSO optimization algorithm into SuperDiMP, KYS^41^, DiMP^26^, and PrDiMP^40^ tracking algorithms to evaluate its performance. The evaluation results are shown in Fig. 3. After incorporating the BRSO optimization algorithm, the SuperDiMP algorithm achieved improvements in success rate, normalized precision, and precision by 1.6%, 2.5%, and 2.5%, respectively. Additionally, the BRSO optimization algorithm demonstrated performance gains across various other tracking architectures. Specifically, the success rate of the DiMP algorithm was improved by 1.3% and its precision was improved by 2%. The PrDiMP algorithm improved its success rate by 0.9% and normalized precision by 0.8%. For the KYS tracker, the success rate improved by 0.4% on the VOT2018 dataset after applying the BRSO optimization algorithm. In summary, the BRSO optimization algorithm exhibited outstanding performance across the aforementioned trackers, effectively enhancing their tracking capabilities.

**Fig. 4:**
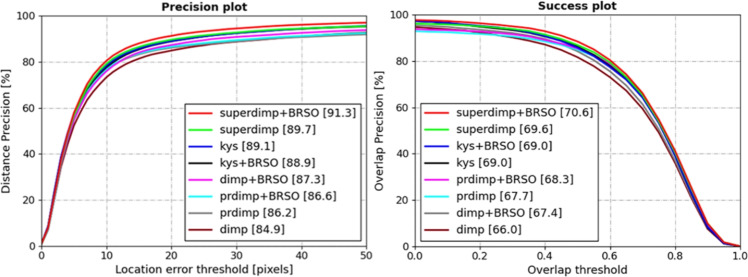
Precision and success rate evaluation results of various algorithms on the OTB-100 dataset.

**OTB-100**^59^: The OTB-100 dataset contains 100 video sequences representing common tracking scenarios. On this dataset, the BRSO optimization algorithm was integrated into four representative trackers: the KYS^41^, DiMP^26^, PrDiMP^40^ and SuperDiMP, for comparative evaluation. Figure 4 illustrates the evaluation results. Notably, the most significant improvement was observed with the DiMP tracker, where the success rate increased by 1.4% and the precision improved by 2.4% after incorporating the BRSO. Similarly, the SuperDiMP tracker’s success rate rose from 69.7% to 70.7%, and accompanied by a 1.6% gain in precision. For PrDiMP, a 0.6% improvement in success rate and a 0.4% increase in precision were recorded. In conclusion, on the OTB-100 dataset, the BRSO optimization algorithm yielded moderate improvements in the performance metrics of the aforementioned trackers.

**Table 1:** Success rate, precision, and normalized precision evaluation results of various algorithms on the Temple-color128 Dataset.

	superdimp	superdimp_BRSO	kys	kys_BRSO	dimp	dimp_BRSO	prdimp	prdimp_BRSO
Success	61.5	*64*	59.8	61.6	60.3	61.5	58.7	60.3
Precision	81.1	*84.1*	78.6	80.5	80.8	81.1	75.5	77.9
Norm-precision	74.4	*78.2*	73	74.6	74	74.2	70.9	73.1

**Temple-color128**^62^: The Temple-Color128 dataset, released by Temple University, consists of 128 video sequences. In this experiment, the proposed BRSO optimization algorithm was integrated into the DiMP^26^, KYS^41^, PrDiMP^40^, and SuperDiMP frameworks for evaluation. The evaluation results are summarized in Table 1. On this dataset, the BRSO optimization algorithm demonstrated significant improvements over the baseline optimization methods in the aforementioned tracking frameworks. For instance, the SuperDiMP tracker showed a 2.5% increase in success rate, a 3% improvement in precision, and a 3.8% boost in normalized precision, leading to a substantial enhancement in tracking performance. The algorithm also delivered notable performance gains in the KYS framework, with a 1.8% rise in success rate, a 1.9% increase in precision, and a 1.6% improvement in normalized precision. For the other two frameworks, DiMP and PrDiMP, the success rates increased by 1.2% and 1.6%, respectively. In summary, the BRSO algorithm achieved consistent and significant performance improvements on the Temple-Color128 dataset, significantly improving the performance metrics of the evaluated trackers.

**Fig. 5:**
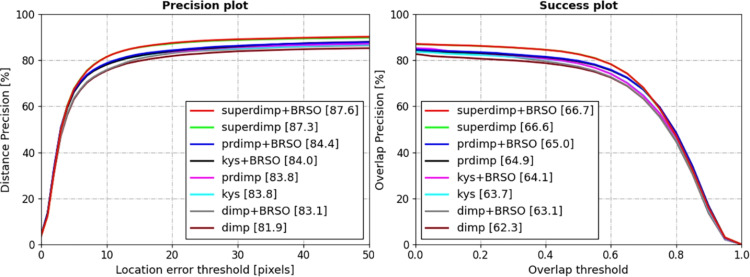
Precision and success rate evaluation results of various algorithms on the UAV123 dataset.

**UAV123**^60^: The UAV123 dataset, consisting of 123 high-definition video sequences captured from a low-altitude aerial perspective, was used to evaluate the proposed BRSO optimization algorithm. In this experiment, the algorithm was integrated into the KYS^41^, SuperDiMP, PrDiMP^40^, and DiMP^26^ trackers for performance evaluation. The evaluation metrics and results are presented in Fig. 5. On this dataset, while the BRSO optimization algorithm did not demonstrate substantial improvements over the aforementioned trackers, it still achieved measurable gains. Specifically, both success rate and accuracy were enhanced by over 0.1%.

### Ablation study

To analyze the roles of the dense sampling module and the random sampling module within the BRSO framework, ablation experiments were conducted on the OTB-100^59^ dataset. The complete BRSO structure was taken as the baseline and integrated into the SuperDiMP algorithm. Comparative analyses were then performed by selectively removing either the dense sampling module or the random sampling module. The experimental results are shown in Table 2.

**Table 2:** Ablation Results of Dense Sampling and Random Sampling.

	Success	Precision	Norm-precision
Superdimp+BRSO	70.6	91.3	85.7
Superdimp+BRSO_Dense	70.2	91.2	85.5
Superdimp+BRSO_Rand	70.2	90.8	85.5

**Analysis of the Dense Sampling Module:** SuperDiMP+BRSO_Dense retains the original bounding box refinement module, while SuperDiMP+BRSO introduces the dense sampling strategy from the BRSO framework. The experimental results demonstrate that SuperDiMP+BRSO outperforms SuperDiMP+BRSO_Dense in both success rate and normalized precision. The dense sampling module effectively enhances the global search capability in bounding box estimation. It achieves this by increasing the spatial coverage and diversity of candidate samples, which helps reduce the risk of falling into local optima.

**Analysis of the Random Sampling Module:** In SuperDiMP+BRSO_Rand, the random sampling module was removed while retaining the dense sampling module. Compared with SuperDiMP+BRSO, the success rate decreased by 0.4%, precision decreased by 0.5%, and normalized precision declined 0.2%. These results demonstrate that the random sampling module critically refines candidate bounding boxes during optimization by mitigating convergence instability, thereby enabling more accurate and stable predictions.

### Convergence analysis of MAP

To verify the optimization behavior of the proposed object state optimization algorithm in multi-frame object tracking tasks, we further analyze its performance under different sampling numbers and iteration settings. In this experiment, the BRSO optimization algorithm is integrated into the SuperDiMP tracking framework and evaluated on the OTB-100 dataset. By default, the number of samples is set to 50 and the maximum number of iterations to 10. The experimental results are summarized in Table 3 and Table 4.

**Table 3:** Tracking performance under different random sampling sizes (m).

	m=30	m=50	m=70	m=100	m=120	m=150	m=200	m=300
Success	70.1	70.6	70.6	70	*70.7*	69.3	70.1	69
Precision	91.1	91.3	91.6	90.9	*91.9*	90	91.2	89.6
Norm-precision	85.3	*86.2*	85.9	85	85.9	83.9	85.2	83.6
FPS	*21*	20	17	16	15	14	12	9

**Table 4:** Tracking performance under different optimization iterations (iter).

	iter=3	iter=5	iter=7	iter=9	iter=10	iter=13	iter=15
Success	69.8	69.9	70.2	*70.7*	70.6	*70.7*	70
Precision	91	90.1	90.7	*91.8*	91.3	91.5	90.7
Norm-precision	85.2	85.1	85.4	*86.1*	85.7	*86.1*	85.5
FPS	35	29	15	20	20	17	15

The experimental results indicate that increasing the number of samples leads to an overall improvement in both success rate and precision, with performance peaking at $m = 120$ before slightly declining. This suggests that excessive sampling may introduce noise or redundant particles, thereby affecting the stability of bounding box estimation. Moreover, a higher sampling number significantly reduces the frame rate, which drops to only 9 FPS at $m = 300$, highlighting substantial computational overhead. When the number of samples is fixed, the number of optimization iterations also influences performance. The best results are observed around $iter = 9$, beyond which performance begins to slightly degrade. The normalized precision remains relatively stable across different settings, while the frame rate consistently decreases as the number of iterations increases. In summary, the BRSO optimization process exhibits a clear convergence trend within an appropriate parameter range, yet reveals a notable trade-off between accuracy and computational efficiency. Therefore, careful selection of sampling number and iteration count is essential for improving the stability and practical applicability of the algorithm.

## Conclusions

This paper proposes an object state optimization algorithm based on Bayesian random sampling for visual object tracking, aimed at improving object state estimation and bounding box optimization. By using dense sampling, the framework enhances the diversity of samples, effectively mitigating the limitations of the optimization algorithm and achieving global optimization. The hybrid architecture, combining Bayesian random sampling with gradient ascent, increases the diversity and randomness of candidate boxes, enabling more accurate object state predictions with fewer optimization steps. Experiments demonstrate that the optimized framework improves the success rate of object bounding box prediction on four challenging benchmark datasets, validating the effectiveness and compatibility of the BRSO optimization algorithm.

## Data Availability

The datasets generated during and/or analysed during the current study are available from the corresponding author on reasonable request.
